# Supplementary material for: Spider venom-derived peptide induces hyperalgesia in Nav1.7 knockout mice by activating Nav1.9 channels
Source: Nat Commun. 2020 May 8;11:2293. doi: 10.1038/s41467-020-16210-y (PMC7210961; doi:10.1038/s41467-020-16210-y)
Supplement: Supplementary file 1 — Supplementary Information [file 41467_2020_16210_MOESM1_ESM.pdf]

Supplementary Information

Spider venom derived peptide induces hyperalgesia in $Na_v1.7$ knockout mice by activating $Na_v1.9$ channel

Zhou et al.

This file includes: Supplementary Figures 1-6, Supplementary Methods and Supplementary Tables 1-12

Supplementary Figures

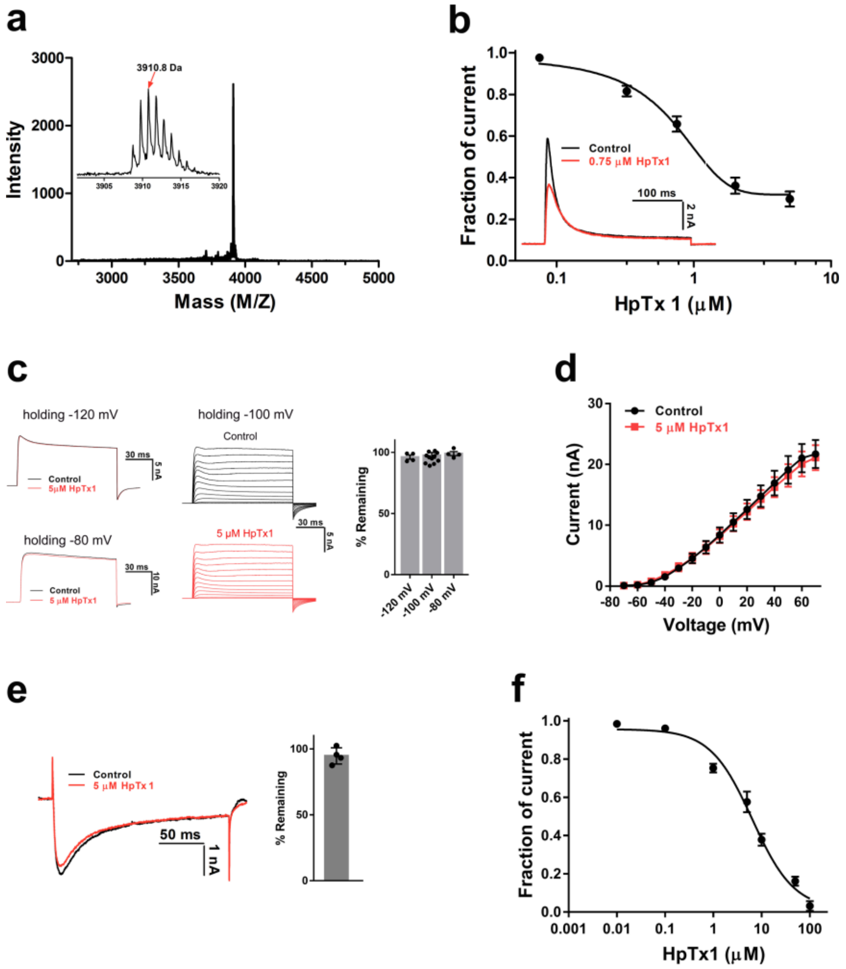

Supplementary Figure 1. (a) The molecular weight of HpTx1 is 3910.8 Da (M+H^{+}), determined by MALDI–TOF/TOF MS. **(b)** Dose-dependent inhibitory curves show the effect of HpTx1 on K_v4.2 (n=5). The inset shows representative current traces in the presence (red) or absence (black) of 0.75 μM HpTx1. **(c)** (*left*) Representative traces show that the outward K^+ currents of mouse DRG neurons are unaffected by 5 μM HpTx1. The currents evoked by depolarization to +20 mV from different holding potentials of -120 mV (*top*) and -80 mV (*bottom*), respectively. (*middle*) Outward DRG K^+ Current traces are shown before (*top*) and after (*bottom*) the application of 5 μM HpTx1. Currents were elicited by 150 ms depolarizing steps to various potentials ranging from -70 mV to +70 mV at increments of +10 mV from a holding potential of -100 mV. (*right*) Scatter plot shows the remaining current after the application of 5 μM HpTx1 at different holding potentials (-80 mV, n=4; -100mV, n=12; -120 mV, n=4). **(d)** Showing no effect on the current-voltage curves (n=12). DRG neurons were held at -100 mV, and families of K^+ currents were induced by 150-ms depolarizing steps to various potentials ranging from-70 mV to +70 mV in 10-mV increments. **(e)** Representative traces (*left*) show that the inward Ca^{2+} currents of DRG neurons are unaffected by 5 μM HpTx1, as also revealed by scatter plots (*right*, n=4). **(f)** Dose-dependent inhibitory curves show the effect of HpTx1 on rNa_v1.6 (n=6). Data are presented as the mean ± S.E.M. Source data are provided as a Source Data file.

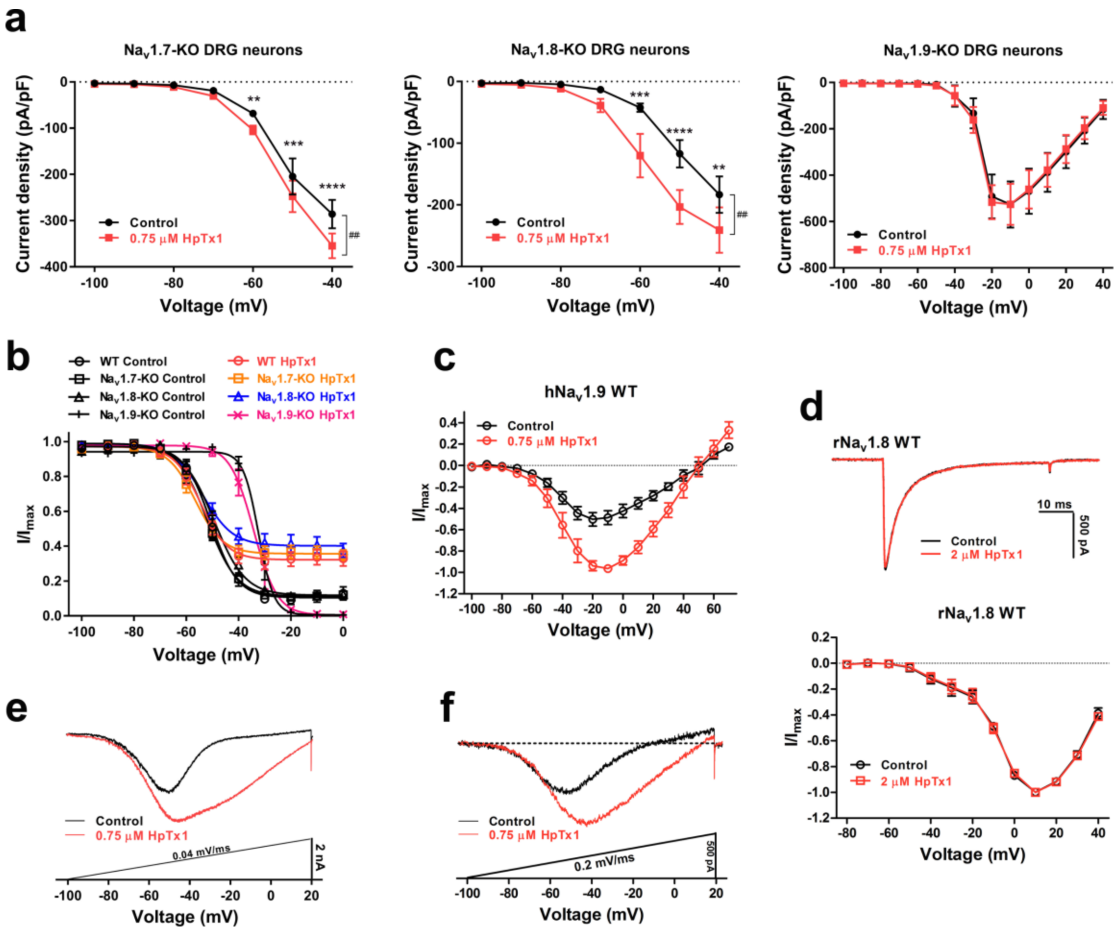

Supplementary Figure 2. **(a)** HpTx1 (0.75 μM) increases the current density of the TTX-R channels in $Na_v1.7$-KO (*left,* n=5, two-way repeated measures ANOVA followed by Bonferroni's multiple comparisons test, Treatment × Voltage: $F_{(6,\ 24)}=9.202$, $P<0.0001$; Treatment: $F_{(1,\ 4)}=26.01$, $^{\#\#}P=0.0070$; Voltage: $F_{(6,\ 24)}=65.43$, $P<0.0001$) and $Na_v1.8$-KO (*middle,* n=6, two-way repeated measures ANOVA followed by Bonferroni's multiple comparisons test, Treatment × Voltage: $F_{(7,\ 35)}=4.772$, $P=0.0008$; Treatment: $F_{(1,\ 5)}=45.77$, $^{\#\#}P=0.0011$; Voltage: $F_{(7,\ 35)}=65.43$, $P<0.0001$) small DRG neurons but not in $Na_v1.9$-KO (*right*) small DRG neurons. **(b)** The effect of 0.75 μM HpTx1 on the steady-state inactivation (SSI) of multiple mouse TTX-R channels (n=7 for WT mice, n=11 for $Na_v1.7$-KO mice, n=12 for $Na_v1.8$-KO mice and n=4 for $Na_v1.9$-KO mice). **(c)** The current-voltage curves of WT $Na_v1.9$ before (black circles) and after (red circles) application of 0.75 μM HpTx1 (n=5). **(d)** The $Na_v1.8$ currents are unaffected by 2 μM HpTx1. Representative current traces (*top*) from ND7/23 cells expressing $rNa_v1.8$ in the absence (black) and presence of 2 μM HpTx1 (red). The current-voltage curves (*bottom*) before (black circles) and after (red squares) application of 2 μM HpTx1 (n=3). **(e-f)** Compared with control treatment, 0.75 μM HpTx1 significantly enhances the ramp currents of TTX-R channels in small DRG neurons of WT mice (**e**, n=4) and $hNa_v1.9$ expressed in ND7/23 cells (**f**, n=6). Data are presented as the mean ± S.E.M. Exact *P* (a) are presented in Supplementary Data 1. Source data are provided as a Source Data file.

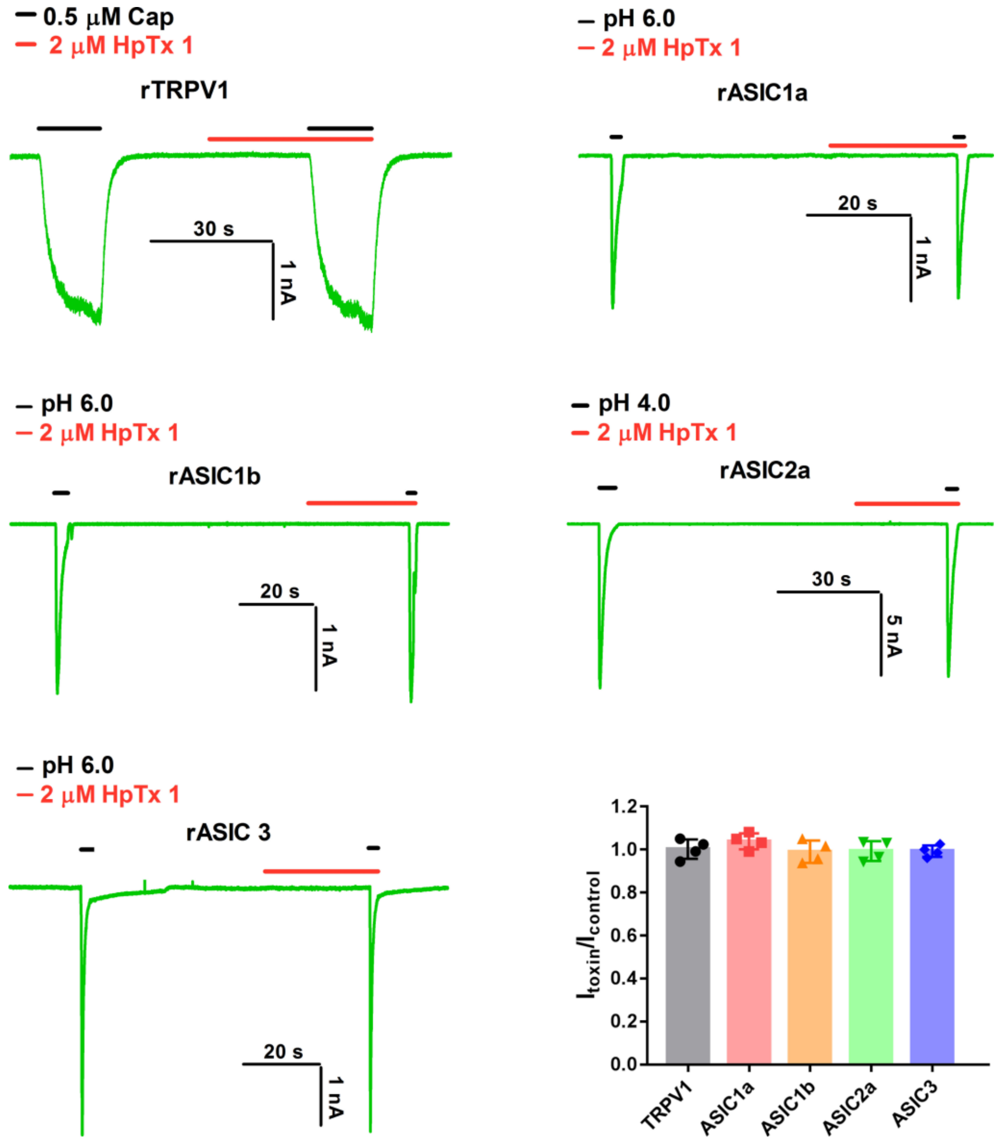

Supplementary Figure 3. HpTx1 has no effect on TRPV1 or ASICs. rTRPV1, rASIC1a, rASIC1b, rASIC2a and rASIC3 channels were expressed in HEK293T cells and were activated by their respective ligands (capsaicin or proton). HpTx1 (2 μM) was perfused continuously before (30 s) and during 0.5 μM capsaicin (TRPV1) or pH stimulation (ASICs). No significant activation or inhibition was observed. Cells were held at -60 mV (n=4). Data are represent the mean ± S.E.M. Source data are provided as a Source Data file.

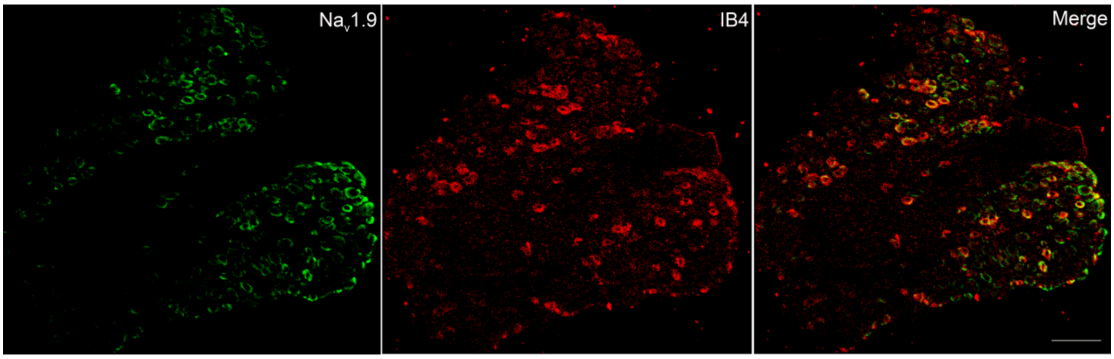

Supplementary Figure 4. Immunoreactivity staining of a DRG section from a WT mouse shows the overlap between $Na_v1.9$ (green) and IB4 (red) in three independent experiments. Scale bar, 100 μm. Source data are provided as a Source Data file.

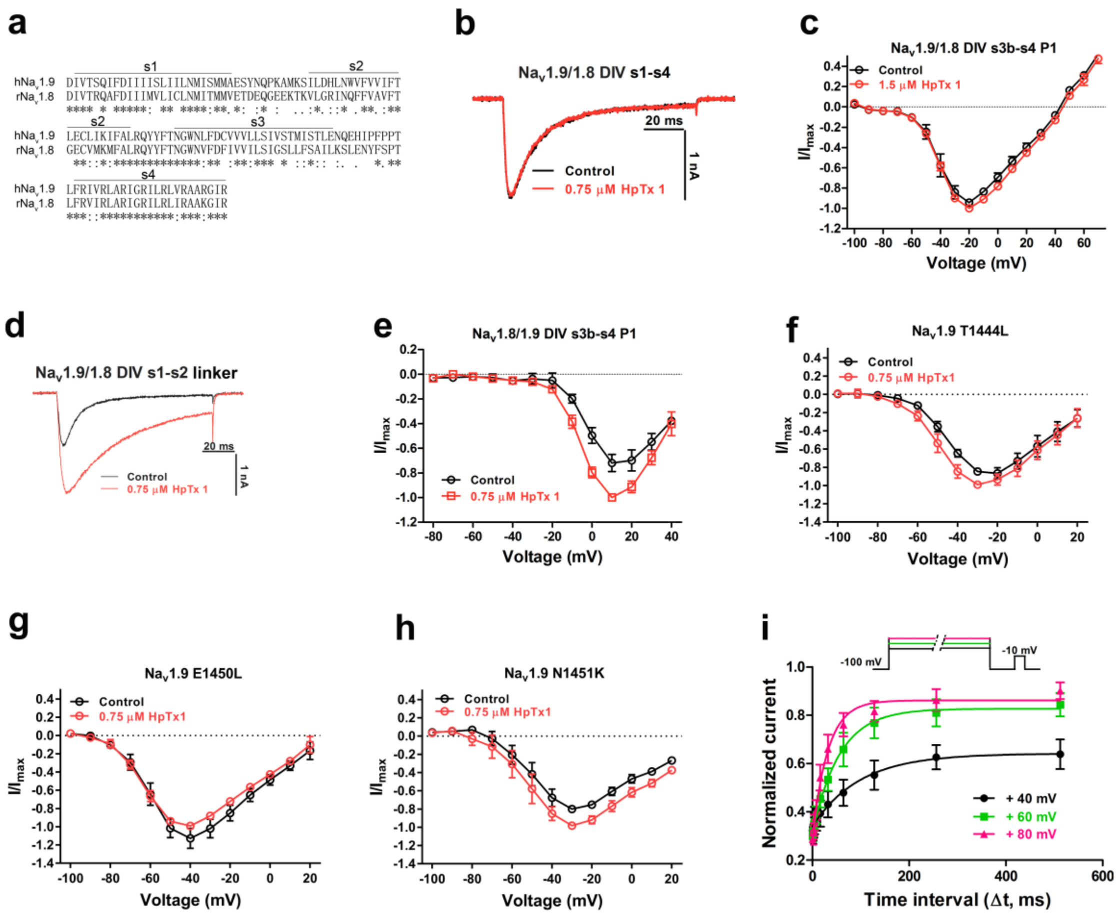

Supplementary Figure 5. (a) Sequence alignment of the hNa$_v$1.9 and rNa$_v$1.8 DIV s1-s4 regions. **(b)** Representative traces show that the chimaera channel Na$_v$1.9/1.8 DIV s1-s4 currents are unaffected by 0.75 μM HpTx1. **(c)** The current-voltage curves of the chimaera channel Na$_v$1.9/1.8 DIV s3b-s4 P1 before (black circles) and after (red circles) application of HpTx1 (n=3). **(d)** Representative current traces from ND7/23 cells expressing the chimaera channel Na$_v$1.9/1.8 DIV s1-s2 linker in the absence (black) and presence of 0.75 μM HpTx1 (red). **(e-h)** The current-voltage curves of chimaera channels Na$_v$1.8/1.9 DIV s3b-s4 P1 (**e**, n=3), Na$_v$1.9 T1444L (**f**, n=3), Na$_v$1.9 E1450L (**g**, n=5) and Na$_v$1.9 N1451K (**h**, n=3) mutant channels before (black circles) and after (red circles) application of 0.75 μM HpTx1. **(i)** Time course of dissociation of 2 μM HpTx1 from hNa$_v$1.7 at 80 mV (τ= 33.3 ± 5.8 ms, n=3), 60 mV (τ= 62.4 ± 12.9 ms, n=4), and 40 mV (τ= 220.3 ± 66.1 ms, n=4). The inset shows the pulse protocol for measuring the rate of toxin dissociation. Cells expressing hNa$_v$1.7 were incubated in HpTx1 for 1 min at a holding potential of -100 mV to allow binding. The rate of toxin dissociation was determined with the illustrated pulse paradigm by stepping to a depolarizing pulse of 80, 60, or 40 mV for 0-512 ms, returning to -100 mV for 100 ms to allow recovery from fast inactivation, and then assessing the effect of the depolarizing pulse with a 50 ms test pulse to -10 mV. Data are presented as the mean ± S.E.M. Source data are provided as a Source Data file.

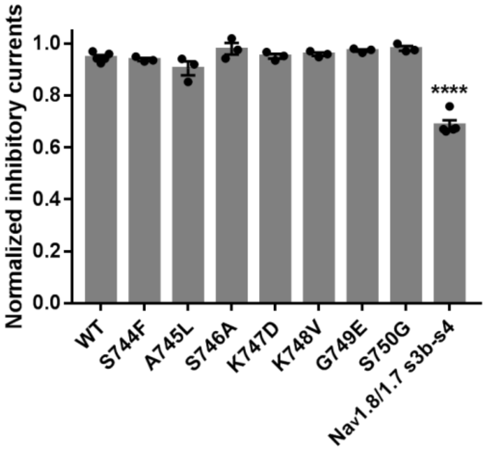

Supplementary Figure 6. The effect of 2 μM HpTx1 on rNa$_v$1.8 WT (n=4), point mutant channels (n=4) and the Na$_v$1.8/1.7 DII s3b-s4 chimaera channel. The point mutant channels were derived from the substitution of the residues at DII s3b-s4 of rNa$_v$1.8 with the corresponding residues of rNa$_v$1.7. The inhibition of HpTx1 on the indicated channel was normalized to that of rNa$_v$1.8. One-way ANOVA with Dunnett's Multiple Comparison Test and compared with WT, $F_{(8, 22)}$=52.37, P<0.0001. Na$_v$1.8/1.7 s3b-s4 vs WT, $^{****}P$=0.0001. Data are represent the mean ± S.E.M. Source data are provided as a Source Data file.

Supplementary Methods

The $Na_v1.9/1.8$ DIV s1-s4, $Na_v1.9/1.8$ DIV s1-s2, $Na_v1.9/1.8$ DIV s3b-s4 P1, $Na_v1.8/1.9$ DIV s3b-s4 P1 and $Na_v1.7/1.8$ DII s3b-s4 chimaeras were constructed by using a recombination strategy. Briefly, for the construction of $Na_v1.9/1.8$ DIV s1-s4 chimaera, the DIV s1-s4 of $Na_v1.8$ was amplified by PCR using a pair of primers ($Na_v1.8$ DIV s1-s4 Fragment Forward: 5’-TTCGACATAGTCACAAGGCAAGCCTT TGACATC-3’, $Na_v1.8$ DIV s1-s4 Fragment Reverse: 5’-TCTGAAGAGCGTCGGGG AGAAGTAG-3’) with their 5’ end extended by a 15 bp long joint which is homologous or reverse compliment to the upstream or downstream flanking sequence of DIV s1-s4 of $Na_v1.9$. A pair of oppositely directed primers ($Na_v1.9$ DIV s1-s4 Vector Forward: 5’-CCGACGCTCTTCAGAATTGTCCGCTTG-3’, $Na_v1.9$ DIV s1-s4 Vector Reverse: 5’-TGTGACTATGTCGAACACGAGACCTTG-3’) was used to linearize the whole $Na_v1.9$ cloned plasmid with the DIV s1-s4 of $Na_v1.9$ deleted. The PCR amplified segment and the linearized plasmid were subjected to 1% agarose gel electrophoresis, respectively. The corresponding bands were recycled using a DNA gel extraction kit (Sangon biotech) and ligated using the Trelief™ SoSoo Cloning Kit (TSINGKE, Beijing, China). Before being transformed to *E.coli* DH5α competent cell, the ligated product was subjected to FastDigest DpnI (Thermo Fisher Scientific, Waltham, MA, USA) treatment at 37 ℃ for 1 hour to remove the template plasmid. All site mutations of $hNa_v1.9$ and $hNa_v1.7$ were constructed by using the QuikChange II XL Site-directed Mutagenesis kit (Agilent Technologies) according to the manufacture’s instruction. All mutations were verified by DNA sequencing. The

swapped regions in chimeric channels were showed in Fig.6a, d and Supplementary Fig.5a. Primers used in this study were described in Supplementary Tables 4-12.

For voltage-clamp recording, the extracellular solution contained (in mM) 150 NaCl, 2 KCl, 1.5 $CaCl_2$, 1 $MgCl_2$, 10 HEPES (pH 7.4 with NaOH) was used. 1μM tetrodotoxin (TTX) was supplemented to block endogenous Na^+ currents in ND7/23 cells. For recording the TTX-R Na^+ currents in DRG neurons, the extracellular solution was supplemented with 1μM tetrodotoxin (TTX) and 0.1 mM $CdCl_2$ to block endogenous TTX-S Na^+ currents and Ca^{2+} currents, respectively. The pipette solution contained (in mM) 35 NaCl, 105 CsF, 10 EGTA, 10 HEPES (pH 7.3 with CsOH). For recording TTX-S Na^+ currents of small DRG neurons, the bath solution contained (in mM) 30 NaCl, 1 $MgCl_2$, 1.8 $CaCl_2$, 5 CsCl, 5 KCl, 25 D-glucose, 5 HEPES, 0.1 $CdCl_2$ and TEA-Cl (pH 7.3 with NaOH), and the pipette solution contained 135 mM CsCl, 10 mM NaCl and 5 mM HEPES (pH 7.3 with CsOH). For recording voltage-gated Ca^{2+} channels in DRG neurons, the bath solution contained (in mM) 130 choline chloride, 25 TEA-Cl, 3 KCl, 5 $BaCl_2$, 0.6 $MgCl_2$, 10 HEPES, 10 D-glucose, and 0.001 TTX (pH 7.3 with NaOH), and the pipette solution contained (in mM) 140 CsCl, 10 EGTA, 0.1 $CaCl_2$, 2 $MgCl_2$, 10 HEPES, and 2 ATP (pH 7.3 with Tris). For recording the voltage-gated K^+ currents in DRG neurons, the bath solution contained (in mM): 130 choline chloride, 5 KCl, 2 $MgCl_2$, 2 $CaCl_2$, 10 HEPES, 10 D-glucose and 0.001 TTX, (pH 7.3 with Tris), and the pipette solution contained (in mM) 120 KCl, 20 NMG, 10 EGTA, 2 Mg-ATP, 10 HEPES, and 0.5 GTP (pH 7.3 with KOH). For recording $K_V4.2$ currents in HEK293 cells, the bath solution

contained (in mM) 140 NaCl, 2 KCl, 1.5 $CaCl_2$, 1 $MgCl_2$, 10 HEPES (pH 7.4 with NaOH), and the pipette solution contained (in mM) 140 KCl, 2.5 $MgCl_2$, 10 HEPES, and 10 EGTA (pH 7.4 with NaOH). For recording TRPV1 and ASICs currents in HEK293 cells, the bath solution contained (in mM) 140 NaCl, 5 KCl, 1 $MgCl_2$, 5 EGTA, 10 D-glucose, 10 mM HEPES (pH 7.4 with NaOH), and the pipette solution contained (in mM)140 KCl, 5 NaCl, 1 $MgCl_2$, 10 EGTA, and 10 HEPES (pH 7.4 with KOH).

For current-clamp recording, the extracellular solution contained (in mM) 140 NaCl, 3 KCl, 2 $CaCl_2$, 2 $MgCl_2$, and 10 HEPES (pH 7.3 with NaOH); the pipette solution contained (in mM) 140 KCl, 0.5 EGTA, 5 HEPES and 2 Mg-ATP (pH 7.3 with KOH). All salts were obtained from Sigma.

To generate activation curves, cells were held at -120 mV or -90 mV and stepped to potentials of -100 to +50 mV in 10-mV or 5-mV increments for 100 ms or 50 ms every 5 s. The G-V curves were obtained by calculating the conductance (G) at each voltage (V) using the equation $G=I/(V-V_{rev})$, with V_{rev} being the reversal potential determined for each cell individually. G-V curves were fitted using a Boltzmann equation: $y = 1/ (1 + exp[(V_{1/2} - V)/\kappa])$ in which $V_{1/2}$, V and κ represent midpoint voltage of kinetics, test potential and slope factor, respectively.

Voltage dependent steady-state inactivation was measured with a series of 500-ms pre-pulses (-120 to 0 mV in 10 mV or 5 mV increments), followed by a 50-ms depolarization to -30 mV (Na_v1.9) or -10 mV (Na_v1.7) to assess the available non-inactivated currents, and the repetition interval was 15 s or 5 s. Peak inward

currents at the test pulse were normalized to the maximal inward current and fit with a Boltzmann function: $I / I_{max} = A + (1 - A) / \{1 + exp[(V - V_{1/2}) / \kappa]\}$, where V represents the inactivating pre-pulse potential, $V_{1/2}$ is the midpoint of the steady-state inactivation, A is the minimal channel availability, and κ is the slope factor.

The ramp current of $hNa_V1.9$ was measured by a small slow ramp depolarization protocol, which was started from the holding potential of -100 mV and steadily increased to 20 mV over 600 ms at the rate of 0.2 mV ms^{-1}. The ramp currents in mouse DRG neurons were recorded by applying a 3 s depolarization ramp potential from -100 to -20 mV under the holding potential of −90 mV. The repetition interval was 10 s.

Dose response curves of HpTx1 were fitted using the following Hill logistic equation: $y=f_{max} - (f_{max} - f_{min})/(1+(x/EC_{50})^n)$ or $y=f_{max} - (f_{max} - f_{min})/(1+(x/IC_{50})^n)$, where f_{max} and f_{min} represent the maximum and minimum response of channel to HpTx1, respectively, and f_{min} was set to 0; x represents HpTx1 concentration and n is an empirical Hill coefficient.

Supplementary Tables

Supplementary Table1. The effects of HpTx1 on AP properties of small DRG neurons

	Control				0.75 μM HpTx1			
	RMP (mV)	Rheobase (pA)	AP amplitude (mV)	Input resistance (MΩ)	RMP (mV)	Rheobase (pA)	AP amplitude (mV)	Input resistance (MΩ)
**WT**	-50.4 ± 1.2 (n=30)	41.3 ± 3.9 (n=30)	117.8 ± 1.3 (n=30)	551.7± 59.8 (n=20)	-48.4 ± 1.3**** (n=30)	32.0 ± 3.4** (n=30)	116.8 ± 1.4 (n=30)	575.6± 72.0 (n=20)
**fNa$_v$1.7**	-48.0 ± 2.4 (n=11)	40.8 ± 4.9 (n=13)	118.7 ± 2.8 (n=11)	667.7± 85.4 (n=11)	-46.1 ± 2.2* (n=11)	30.8 ± 4.3* (n=13)	117.4 ± 2.4 (n=13)	648.2± 65.5 (n=11)
**Na$_v$1.7-KO**	-49.6 ± 1.0 (n=28)	48.3 ± 4.6 (n=29)	108.2 ± 1.3# (n=29)	669.7± 77.5 (n=17)	-46.4 ± 1.1** (n=28)	31.7 ± 3.4*** (n=29)	106.5 ± 1.3 (n=29)	649.7± 54.8 (n=17)
**Na$_v$1.8-KO**	-46.9 ± 1.9 (n=18)	40.0 ± 6.1 (n=18)	83.4 ± 3.2#### (n=18)	697.1± 66.9 (n=16)	-44.3 ± 1.8** (n=18)	38.9 ± 8.0 (n=18)	82.5 ± 2.3 (n=18)	813.1± 75.1 (n=16)
**Na$_v$1.7/Na$_v$1.8-DKO**	-52.3 ± 1.4 (n=19)	60.0 ± 6.3 (n=19)	88.4 ± 2.3#### (n=19)	709.3± 81.3 (n=14)	-47.1 ± 1.2**** (n=19)	48.4 ± 6.4* (n=19)	87.0 ± 2.5 (n=19)	723.0± 57.9 (n=14)
**Na$_v$1.9-KO**	-50.2 ± 1.7 (n=29)	40.0 ± 4.3 (n=25)	110.6 ± 1.3 (n=25)	685.3± 40.9 (n=26)	-49.1 ± 1.7 (n=29)	50.4 ± 5.6** (n=25)	109.7 ± 1.7 (n=25)	778.6± 51.8 (n=26)

*P< 0.05, **P< 0.01, ***P< 0.001, ****P< 0.0001 when compared with Control.

#P< 0.05, ####P< 0.0001, when compared with fNa$_v$1.7.

n is presented as the number of the separate experimental cells.

two-way ANOVAs with Tukey's multiple comparisons test was used. F, P values and df (degrees of freedom) are presented in Supplementary Data 1.

Supplementary Table 2. The changed of Rheobase of DRG neurons after 0.75 μM HpTx1 treatment.

	Decreased	Unchanged	Increased
**WT**	50% (15/30)	40% (12/30)	10% (3/30)
**fNa$_v$1.7**	61.5% (8/13)	30.8% (4/13)	7.7% (1/13)
**Na$_v$1.7-KO**	65.5% (19/29)	24.1%（7/29）	10.3% (3/29)
**Na$_v$1.9-KO**	8% (2/25)	44% (11/25)	48% (12/25)
**Na$_v$1.8-KO**	22.2% (4/18)	66.7% (12/18)	11.1% (2/18)
**Na$_v$1.7/Nav1.8-DKO**	42.1% (8/19)	52.6% (10/19)	5.3% (1/19)

Supplementary Table 3. The effects of HpTx1 on activation and inactivation of $Na_v1.7$ and $Na_v1.9$.

	Control						0.75 μM HpTx1					
	Voltage dependence of Activation (mV)			Voltage dependence of Inactivation (mV)			Voltage dependence of Activation (mV)			Voltage dependence of Inactivation (mV)		
	$V_{1/2}$	k	n	$V_{1/2}$	k	n	$V_{1/2}$	k	n	$V_{1/2}$	k	n
**$Na_v1.7$**	-21.9 ± 1.9	5.2 ± 0.2	4	-70.7 ± 1.9	-4.8 ± 0.1	7	-22.1 ± 3.3	6.9 ± 0.3	4	-73.5 ± 1.7	-4.7 ± 0.1	7
**$Na_v1.9$**	-38.2 ± 3.6	8.9 ± 1.5	6	-69.9 ± 1.3	-9.0 ± 0.7	8	-35.2 ± 3.0	10.0 ± 0.9	6	-56.5 ± 2.4***	-15.0 ± 0.6****	8

***P< 0.001, ****P< 0.0001, when compared with Control.

parametric unpaired two-tailed t-test was used, Control vs HpTx1: $V_{1/2}$ (t_{13}=4.263, P=0.0009), k (t_{13}=6.193, P<0.0001).

n is presented as the number of the separate experimental cells.

Supplementary Table 4: Primers used in this study to construct $Na_v1.9/1.8$ DIV s1-s4 chimaeras.

Primer name	Sequence (5'-3')
**$Na_v1.8$ DIV s1-s4 F for**	TTCGACATAGTCACAAGGCAAGCCTTTGACATC
**$Na_v1.8$ DIV s1-s4 F rev**	TCTGAAGAGCGTCGGGGAGAAGTAG
**$Na_v1.9$ DIV s1-s4 V for**	CCGACGCTCTTCAGAATTGTCCGCTTG
**$Na_v1.9$ DIV s1-s4 V rev**	TGTGACTATGTCGAACACGAGACCTTG

Supplementary Table 5: Primers used in this study to construct $Na_v1.9/1.8$ DIV s1-s2 linker chimaeras.

Primer name	Sequence (5'-3')
**$Na_v1.8$ DIV s1-s2 linker F for**	ATAAGTCTCATTATCCTCAACATGATCACCA
**$Na_v1.8$ DIV s1-s2 linker F rev**	GACCACAAAGACCCAGTTGATTCTGCCCAGAAC
**$Na_v1.9$ DIV s1-s2 linker V for**	TGGGTCTTTGTGGTCATCTTTAC
**$Na_v1.9$ DIV s1-s2 linker V rev**	GATAATGAGACTTATGATG

Supplementary Table 6: Primers used in this study to construct $Na_v1.9/1.8$ DIV s3b-s4 P1 chimaeras.

Primer name	Sequence (5’-3’)
**$Na_v1.8$ DIV s3b-s4 P1 F for**	TTATTTGACTGTGTGGTCGTGCTTCTGTCCATTGTGAGTCTGCTGT
**$Na_v1.8$ DIV s3b-s4 P1 F rev**	CGGAGGGAAAGGAATGTGCTCCTGCTTAAGGATTGCAGAAAAC
**$Na_v1.9$ DIV s3b-s4 P1 V for**	AAGCACGACCACACAGTCAAATAAA
**$Na_v1.9$ DIV s3b-s4 P1 V rev**	CAGGAGCACATTCCTTTCCCTC

Supplementary Table 7: Primers used in this study to construct Na$_v$1.8/1.9 DIV s3b-s4 P1 chimaeras.

Primer name	Sequence (5'-3')
**Na$_v$1.9 DIV s3b-s4 P1 F for**	GTGTTCGACTTCATAGTGGTGATCCTTTCCATTGGTAGTAC
**Na$_v$1.9 DIV s3b-s4 P1 F rev**	CGGGGAGAAGTAGTTTTCCAGTGAATTTTCCAAGGTAGAAATC
**Na$_v$1.8 DIV s3b-s4 P1 V for**	GATCACCACTATGAAGTCGAACACG
**Na$_v$1.8 DIV s3b-s4 P1 V rev**	TCACTGGAAAACTACTTCTCCCCGAC

Supplementary Table 8: Primers used in this study to construct $Na_v1.7/1.8$ DII s3b-s4 chimaeras.

Primer name	Sequence (5'-3')
**$Na_v1.8$ DII s3b-s4 F for**	ACTTTAAGTTTAGTGGAGCTGAGTGCATC
**$Na_v1.8$ DII s3b-s4 F rev**	GAGCAGTCTGAATGAACGGAGCACA
**$Na_v1.7$ DII s3b-s4 V for**	CACTAAACTTAAAGTCACAATAAGGC
**$Na_v1.7$ DII s3b-s4 V rev**	TCATTCAGACTGCTCCGAGTCTTC

Supplementary Table 9: Primers used in this study to construct Na_v1.8/1.7 DII s3b-s4 chimaeras.

Primer name	Sequence (5'-3')
**Na_v1.7 DII s3b-s4 F for**	ACCGTGAGCCTTCTGGAGCTCTTTCTAGCAG
**Na_v1.7 DII s3b-s4 F rev**	CAGCAAGCGTAAGGTTCGCAGAACTGACAATCC
**Na_v1.8 DII s3b-s4 V for**	ACCTTACGCTTGCTGCGGGTCTTC
**Na_v1.8 DII s3b-s4 V rev**	CAGAAGGCTCACGGTGACGATG

Supplementary Table 10: Primers used in this study to construct $Na_v1.7$ point mutations.

Primer name	Sequence (5'-3')
**$Na_v1.7$ F813S for**	AGTTTAGTGGAGCTCAGTCTAGCAGATGTG
**$Na_v1.7$ F813S rev**	GAGCTCCACTAAACTTAAAGTCACA
**$Na_v1.7$ L814A for**	TTAGTGGAGCTCTTTGCAGCAGATGTGGA
**$Na_v1.7$ L814A rev**	AAAGAGCTCCACTAAACTTAAAG
**$Na_v1.7$ A815S for**	GTGGAGCTCTTTCTATCAGATGTGGA
**$Na_v1.7$ A815S rev**	TAGAAAGAGCTCCACTAAACTTAAA
**$Na_v1.7$ D816K for**	GAGCTCTTTCTAGCAAAGGTGGAAGGATTG
**$Na_v1.7$ D816K rev**	TGCTAGAAAGAGCTCCACTAAAC
**$Na_v1.7$ V817K for**	CTCTTTCTAGCAGATAAGGAAGGATTGTC
**$Na_v1.7$ V817K rev**	ATCTGCTAGAAAGAGCTCCAC
**$Na_v1.7$ E818G for**	TTTCTAGCAGATGTGGGAGGATTGTCAG
**$Na_v1.7$ E818G rev**	CACATCTGCTAGAAAGAGCTCCACT
**$Na_v1.7$ E818R for**	TTTCTAGCAGATGTGAGAGGATTGTCAGTTC
**$Na_v1.7$ E818R rev**	CACATCTGCTAGAAAGAGCTCCACT
**$Na_v1.7$ G819S for**	CTAGCAGATGTGGAATCATTGTCAGTTCTG
**$Na_v1.7$ G819S rev**	TTCCACATCTGCTAGAAAGAGCTCC

Supplementary Table 11: Primers used in this study to construct Na$_v$1.8 point mutations.

Primer name	Sequence (5'-3')
**Na$_v$1.8 S744F for**	AGCCTTCTGGAGCTGTTTGCATCCAAGAA
**Na$_v$1.8 S744F rev**	CAGCTCCAGAAGGCTCACG
**Na$_v$1.8 A745L for**	CTTCTGGAGCTGAGTCTATCCAAGAAGGG
**Na$_v$1.8 A745L rev**	ACTCAGCTCCAGAAGGCTCACGG
**Na$_v$1.8 S746A for**	CTGGAGCTGAGTGCAGCCAAGAAGGGCAGCCT
**Na$_v$1.8 S746A rev**	TGCACTCAGCTCCAGAAGGCTC
**Na$_v$1.8 K747D for**	GAGCTGAGTGCATCCGATAAGGGCAGCCTG
**Na$_v$1.8 K747D rev**	GGATGCACTCAGCTCCAGAAG
**Na$_v$1.8 K748V for**	CTGAGTGCATCCAAGGTGGGCAGCCTGTC
**Na$_v$1.8 K748V rev**	CTTGGATGCACTCAGCTCCAGAAGG
**Na$_v$1.8 G749E for**	AGTGCATCCAAGAAGGAAAGCCTGTCTGT
**Na$_v$1.8 G749E rev**	CTTCTTGGATGCACTCAGCTCCAGA
**Na$_v$1.8 S750G for**	GCATCCAAGAAGGGCGGCCTGTCTGTGCTC
**Na$_v$1.8 S750G rev**	GCCCTTCTTGGATGCACTCAGC

Supplementary Table 12: Primers used in this study to construct $Na_v1.9$ point mutations.

Primer name	Sequence (5'-3')
**$Na_v1.9$ T1444L for**	CTTTCCATTGTTAGTCTAATGATTTCTACC
**$Na_v1.9$ T1444L rev**	ACTAACAATGGAAAGAAGCACGACC
**$Na_v1.9$ M1445L for**	TCCATTGTTAGTACACTGATTTCTACCTTG
**$Na_v1.9$ M1445L rev**	TGTACTAACAATGGAAAGAAGCACG
**$Na_v1.9$ I1446F for**	ATTGTTAGTACAATGTTTTCTACCTTG
**$Na_v1.9$ I1446F rev**	CATTGTACTAACAATGGAAAGAAGC
**$Na_v1.9$ T1448A for**	AGTACAATGATTTCTGCCTTGGAAAATCAGG
**$Na_v1.9$ T1448A rev**	AGAAATCATTGTACTAACAATGGAAAG
**$Na_v1.9$ L1449I for**	ACAATGATTTCTACCATCGAAAATCAGGAGCAC
**$Na_v1.9$ L1449I rev**	GGTAGAAATCATTGTACTAACAATGGA
**$Na_v1.9$ E1450L for**	ATGATTTCTACCTTGCTAAATCAGGAGCACAT
**$Na_v1.9$ E1450L rev**	CAAGGTAGAAATCATTGTACTAACAATGGA
**$Na_v1.9$ N1451K for**	TTCTACCTTGGAAAAGCAGGAGCACATTCCTTTC
**$Na_v1.9$ N1451K rev**	TTTCCAAGGTAGAAATCATTGTACT
