# Supplementary material for: Spider venom-derived peptide induces hyperalgesia in Nav1.7 knockout mice by activating Nav1.9 channels
Source: Nat Commun. 2020 May 8;11:2293. doi: 10.1038/s41467-020-16210-y (PMC7210961; doi:10.1038/s41467-020-16210-y)
Supplement: Supplementary file 3 — Description of Additional Supplementary Information [file 41467_2020_16210_MOESM3_ESM.pdf]

Description of Additional Supplementary Files

File Name: Supplementary Data 1
Description: Exact p value and related statistical methods.
